# Pterostilbene Ameliorates Fumonisin B1-Induced Cytotoxic Effect by Interfering in the Activation of JAK/STAT Pathway

**DOI:** 10.3390/antiox11122360

**Published:** 2022-11-28

**Authors:** Jian Jin, Yiyi Shan, Liangliang Zhang, Zhengchang Wu, Shenglong Wu, Mingan Sun, Wenbin Bao

**Affiliations:** 1 College of Animal Science and Technology, Yangzhou University, Yangzhou 225009, China; 2 Institute of Comparative Medicine, College of Veterinary Medicine, Yangzhou University, Yangzhou 225009, China

**Keywords:** fumonisin B1, pterostilbene, alveolar macrophage, cytotoxicity, JAK/STAT signaling pathway, pig

## Abstract

Fumonisin B1 (FB1) is a mycotoxin that poses a great threat to agricultural production and the health of humans and animals. Pterostilbene (PTE) is a natural plant polyphenolic compound with good anti-inflammatory, antioxidant and cell regeneration effects, yet its effectiveness in treating FB1-induced cytotoxicity remains to be explored. In this study, we used porcine alveolar macrophages (3D4/21) as a model to characterize the cytotoxicity induced by FB1, and to investigate the potential alleviating effect of PTE on FB1-induced cytotoxicity. We demonstrate that FB1 induces cytotoxicity, apoptosis, pro-inflammatory cytokine production and mitochondrial damage, which can be largely recovered by PTE treatment, suggesting the promising application of PTE to treat FB1-induced damage. Mechanistically, FB1 activates the JAK/STAT signaling pathway, while PTE attenuates FB1-induced cytotoxicity through the inhibition of key JAK/STAT genes such as *JAK2* and *STAT3*. Overall, our study characterized the molecular mechanism for FB1-induced cytotoxicity and found PTE to be a promising component which can alleviate FB1-induced cytotoxicity by interfering in the activation of JAK/STAT pathway.

## 1. Introduction

Fumonisins are a class of water-soluble mycotoxins mainly produced by *Fusarium moniliforme*. Due to their widespread occurrence in crops, Fumonisins are considered to be key food contaminants in a large number of products worldwide [1,2,3]. Among them, Fumonisin B1 (FB1) is internationally classified as a class 2B carcinogen, and poses a great threat to human and animal health [4,5]. Importantly, it is difficult to destroy FB1 through common thermal processing due to its thermal stability; moreover, the high contamination rate, high detection cost and difficult differential diagnosis of FB1 make it difficult to completely eliminate its risk to livestock and poultry through feed [6]. FB1 has been shown specifically to cause pulmonary edema in pigs and leukoencephalomalacia in horses [2,7,8]. Multiple toxic effects, caused by exposure of humans and animals to FB1, have made FB1 contamination a serious public health problem [9]. Currently, the mechanism of toxic effects on FB1 is still not fully understood; it is suspected that it may be caused by disruption of sphingolipid biosynthesis [10]. The development of effective approaches to alleviate or remove FB1 pollution has become an urgent need for not only healthy livestock and poultry breeding, but also human food safety.

Pterostilbene (3,5-dimethoxy-4′-hydroxystilbene, PTE) is a natural stilbene compound that can be isolated from plants such as red sandalwood, blueberry, grape and aloe, and is common in the human diet [11,12]. PTE has the beneficial effects of anti-inflammation, antioxidation and promoting cell regeneration [13,14]. It has been shown that PTE may attenuate oxidative stress and inflammatory responses induced by advanced glycation end products through the RAGE/MAPK/NF-κB pathway [15]. In studies of sepsis in mice, PTE attenuates sepsis-induced liver injury by reducing inflammatory responses and inhibiting hepatocyte apoptosis [16]. Similarly, PTE can attenuate inflammatory responses by reducing the expression of pro-inflammatory cytokines in diabetic mice [17]. PTE attenuates glutamate-induced oxidative stress injury by reducing ROS production in neural cells and increasing SOD and GSH activities through the nuclear factor erythroid 2-related factor 2 signaling pathway [18]. In a fructose-induced T2DM rat model, PTE significantly increased SOD and GSH levels to attenuate hepatic oxidative stress [19]. PTE is also an effective fungicide and fungal spore agent; in vitro experiments have shown that PTE has a killing effect on *Leptosphaeria maculans*, and can significantly reduce the cell viability of hyphae [20]. As an ideal natural compound of plant origin widely existing in nature, it is still unclear whether PTE can reduce the toxic effect of FB1, and through which mechanism.

In this study, we used porcine alveolar macrophages (3D4/21) as a model to study the cytotoxicity induced by FB1, and to explore the mitigating effect of PTE on FB1-induced cytotoxicity. We first analyzed the effects of FB1 induction on cell viability and cell morphology, then analyzed the effects of FB1 induction on apoptosis, inflammation, and mitochondrial damage using quantitative polymerase chain reaction, cell flow cytometry, and enzyme-linked immunosorbent assay. We then further investigated the alleviating effect of PTE on FB1-induced cytotoxicity through salvage experiments. Finally, through transcriptome sequencing, we screened the signal transduction pathways through which FB1 exerts its toxic effects, and verified the effect of PTE on alleviating FB1 cytotoxicity, and its possible mechanism.

## 2. Materials and Methods

### 2.1. Cell Culture

Porcine alveolar macrophages (3D4/21) (ATCC, CRL-2843) were cultured in RPMI-1640 culture medium (Gibco, Grand Island, NY, USA) containing a mixture of 10% fetal bovine serum (FBS) (Gibco, Grand Island, NY, USA) and 1% Pen-Strep (penicillin (100 U/mL), streptomycin (0.1 mg/mL)) (Beijing Solarbio Science & Technology Co., Ltd., Beijing, China). The cells were cultured in a 37 °C incubator with a continuous supply of 5% CO_2_.

### 2.2. Cell Viability Assay

FB1 (≥98%, HPLC) (Aladdin, Shanghai, China) was dissolved in enzyme-free water. 3D4/21 cells were counted using a cell counting plate, and the cell suspension was evenly inoculated in a 96-well plate with 2000 cells per well (100 μL). After being cultured in a CO_2_ incubator at 37 °C for 24 h in RPMI-1640 complete culture medium, the final FB1 concentrations of 0, 10, 20, 30, 40, 50 and 60 μg/mL were replaced. 

PTE (purity 97%) (Aladdin, Shanghai, China) was dissolved in dimethyl sulfoxide (DMSO). 3D4/21 cells were counted using a cell counting plate, and the cell suspension was evenly inoculated into a 96-well plate at 2000 cells (100 μL) per well, then cultured in a 5% CO_2_ incubator at 37 °C for 24 h. An RPMI-1640 complete medium with final PTE concentrations of 0, 1, 2, 4, 6, 8, and 10 μg/mL was replaced, and the DMSO control group was additionally set. Cell viability was detected using Cell Counting Kit-8 (Dojindo Laboratory, Shanghai, China) according to the manufacturer ‘s protocol. CCK-8 solution was added at 10 μL per well in a 96-well plate, and the cell culture plate was tilted during the liquid addition process to avoid bubble interference with optical density (OD) values. The optical density measurements were performed on a Tecan Infinit200 microplate reader (Sunrise, Tecan, Switzerland) at a wavelength of 450 nm.

### 2.3. Immunofluorescence Staining

After the corresponding experimental treatment, 3D4/21 cells were fixed with 4% paraformaldehyde at room temperature for 30 min, treated with 1% Triton X-100 for 10 min, and blocked with bovine serum albumin (Beyotime Institute of Biotechnology, Shanghai, China) at room temperature for 1 h. The samples were then incubated overnight with primary antibody (α-Tubulin antibody, abcam Co., Ltd., Cambridge, MA, USA) at 4 °C. The cells were gently washed with PBST and incubated for 1 h, in the dark, with fluorescently labeled secondary antibodies (abcam Co., Ltd., Cambridge, MA, USA). After gently washing the cells 3 times with PBST, DAPI was added to counterstain cell nuclei. Samples were observed and photographed under a fluorescence microscope (Leica Microsystems, Wetzlar, Germany).

### 2.4. Apoptosis Assessment

3D4/21 cells were cultured in 6-well plates and collected after corresponding experimental treatments, and apoptosis was detected by an Annexin V-FITC/PI kit (Beijing Solarbio Science & Technology Co., Ltd., Beijing, China). This was followed by Annexin V-FITC and PI staining incubations according to the manufacturer ‘s protocol. Apoptosis was detected by flow cytometry (Beckman Coulter, Brea, CA, USA) within 1 h, and CytExpert 2.3 was used for data analysis.

### 2.5. Reactive Oxygen Species Detection

The levels of intracellular reactive oxygen species (ROS) were measured using a reactive oxygen species assay kit (Beyotime Institute of Biotechnology, Shanghai, China). After the 3D4/21 cells were treated with the corresponding experiments, the cell samples were collected and incubated with an appropriate volume of diluted DCFH-DA in a 37 °C cell culture incubator for 20 min, before the cells were washed 3 times using serum-free RPMI-1640 medium to fully remove DCFH-DA that did not enter the cells. The level of intracellular ROS was detected by fluorescence microscope and flow cytometry.

### 2.6. Measurement of the Mitochondrial Membrane Potential

JC-1 is an ideal fluorescent probe for detecting mitochondrial membrane potential (∆Ψm). 3D4/21 cells were cultured in 12-well plates and assayed using the mitochondrial membrane potential assay kit (JC-1) (Beyotime Institute of Biotechnology, Shanghai, China) according to the manufacturer ‘s protocol after corresponding experimental treatments. The cells were observed with a fluorescence microscope, and the ratio of red and green fluorescence intensities detected by flow cytometry represents the quantified ∆Ψm.

### 2.7. Detection of Cell Membrane Integrity

3D4/21 cells were treated with the corresponding experiments and cell membrane integrity was detected using a Calcein/PI Cytotoxicity Assay Kit (Beyotime Institute of Biotechnology, Shanghai, China). Calcein-AM (Calcein) and propidium iodide (PI) probes can detect the activity of intracellular esterase and the integrity of cell membranes, respectively. Calcein-AM itself is not fluorescent and is hydrolyzed by endogenous esterases in living cells to generate calcein, a highly negatively charged polar molecule that does not penetrate the cell membrane, but emits strong green fluorescence. Because dead cells lack esterase, or have very low esterase activity and cannot or rarely produce calcein, only living cells will be stained with strong green fluorescence; dead cells cannot be stained or will be very weakly stained. PI, a nucleic acid red fluorescent dye, can only stain dead cells whose cell membrane integrity is compromised, because it cannot penetrate the cell membrane of living cells.

### 2.8. Lactate Dehydrogenase Assay

Apoptosis or necrosis leads to the release of lactate dehydrogenase (LDH) from the cytoplasm into the culture medium. LDH can be used as an important indicator of cytotoxicity and cell membrane integrity. Quantitative analysis of cytotoxicity can be achieved by detecting the activity of LDH released into the cell culture. The activity of LDH released into culture medium by 3D4/21 cells under FB1, PTE, and their combination was detected using the lactate dehydrogenase (LDH) assay kit (Nanjing Jiancheng Bioengineering Institute, Nanjing, China) according to the manufacturer ‘s protocol.

### 2.9. ELISA Assay

The supernatant of the cell culture of each sample was collected, and the secretion level of cytokines (IL-6, IL-1β, IL-8, TNF-α and IFN-γ) in the supernatant of each cell culture was detected strictly according to the instructions of the ELISA kit (Beijing Solarbio Science & Technology Co., Ltd., Beijing, China).

### 2.10. RNA Extraction and cDNA Synthesis

Total RNA was extracted from the corresponding treated cells using a TRIzol reagent (Invitrogen, Carlsbad, CA, USA). The degree of RNA integrity was detected by 1% formaldehyde denaturing agarose gel electrophoresis, and after the concentration and purity were determined using a ND-1000 nucleic acid/protein concentration tester, it was stored in an ultra-low-temperature freezer at −80 °C until use.

The reverse transcription experiment performed with HiScript Q RT SuperMix for qPCR (+gDNA wiper) (Vazyme Biotech Co., Ltd., Nanjing, China). The total RNA extracted was used as a template for synthesizing cDNA; 10 μL of the reaction system contained 2 μL of 5 × qRT SuperMix II and 500 ng of total RNA, and RNase free ddH_2_O made up 10 μL

### 2.11. qPCR Assay

According to the gene sequence published in the GenBank database, qPCR primers were designed using Primer Premier 5.0 software. The *GAPDH* genes were set as the internal controls. All primer synthesis was conducted by Sangon Biotechnology (Shanghai, China), and the corresponding sequences are shown in (Table S1). qPCR analysis was performed with AceQ Universal SYBR qPCR Master Mix (Vazyme Biotech Co., Ltd., Nanjing, China). All qPCR reactions were conducted in a 20 µL volume composed of 2 µL of cDNA, 0.4 µL of each primer (10 µmol/L), 10 µL of 2 × AceQ Universal SYBR qPCR Master Mix, and 7.2 µL of ddH_2_O. Three independent experimental replicates were conducted for all analyses.

### 2.12. RNA Sequencing

Total RNA was extracted from the experimental samples using the TRIzol (Invitrogen, Carlsbad, CA, USA) kit according to the instructions; RNA purity and concentration were preliminarily detected using a NanoDrop2000 spectrophotometer (Thermo Scientific, Waltham, MA, USA), and RNA integrity was accurately quantified using an Agilent 2100 (Agilent Technologies, Santa Clara, CA, USA) bioanalyzer. 

Then, we removed the rRNAs from the total RNA of the sample, retained the mRNAs and ncRNAs, reverse transcribed the obtained RNA, purified the cDNA fragment using the QiaQuick PCR kit (Qiagen, Venlo, Holland), repaired the end, added PolyA, added sequencing linker, degraded the product by UNG (Uracil-N-Glycosylase) enzyme and amplified the product by PCR, and sequenced the library by Illumina HiSeqTM4000. The raw data on the machine were subjected to quality control and data filtering, and the processed reads were aligned to the pig reference genome (release Sscrofa11.1) using HISAT2 [21]. Differentially expressed genes were identified by DESeq2 [22], while differentially expressed genes were validated by qPCR (Table S2).

### 2.13. Statistical Analyses

The results of relative quantification were analyzed and processed using the 2^−ΔΔCt^ method [23], and the expression levels were normalized to appropriate internal control genes. Data analysis was performed using SPSS 25.0 software, and test data were expressed as mean ± standard deviation (mean ± SD) with multiple replicates for each independent experiment or treatment.

## 3. Results

### 3.1. FB1 Inhibits Cell Viability and Induces Apoptosis and Inflammation

**Figure 1:**
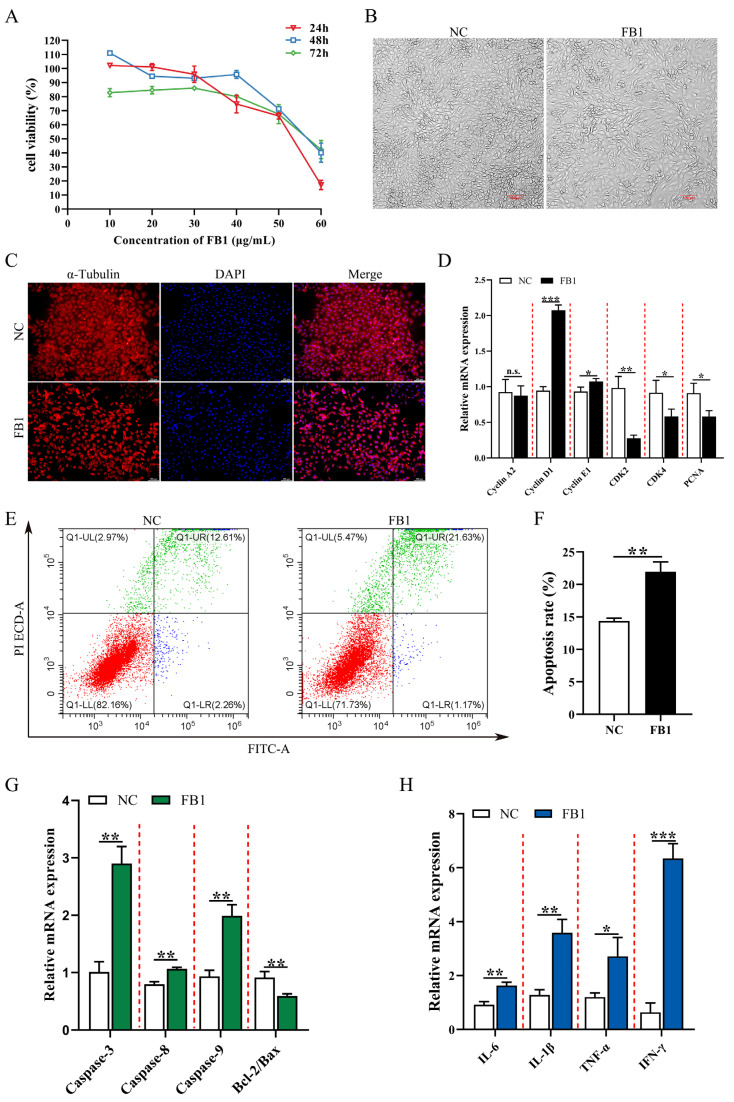
The effects of FB1 on the survival rate and cell morphology of 3D4/21 cells. (**A**) The viability of 3D4/21 cells treated with different concentrations of FB1 (0, 10, 20, 30, 40, 50 and 60 μg/mL) for different times (24, 48 and 72 h). (**B**) Cell morphology of 3D4/21 cells cultured for 24 h in control and 50 μg/mL FB1 treatment groups. Scale bar: 100 µm. (**C**) The changes of cytoskeleton protein α-Tubulin in 3D4/21 cells detected by immunofluorescence. The red fluorescence represents the protein fiber grid structure that binds to the α-Tubulin antibody in the cytoplasm, and the blue fluorescence represents the nucleus stained by DAPI. Scale bar: 100 µm. (**D**) Effect of FB1 induction on the expression of cell cycle-related genes in 3D4/21 cells. (**E**) 3D4/21 cells were treated with 50 μg/mL FB1 for 24 h, stained with Annexin V-FITC and analyzed by flow cytometry. (**F**) Quantification of (**E**); (**G**) Effect of FB1 on the expression of apoptosis-related genes in 3D4/21 cells. (**H**) Effect of FB1 on the expression of inflammation-related genes in 3D4/21 cells. NC: (3D4/21 cells without FB1 exposure), FB1 (50 μg/mL FB1 for 24 h). Data are presented as the mean ± SD; n.s., not significant; * *p* < 0.05; ** *p* < 0.01; *** *p* < 0.001.

In order to investigate the effect of FB1 on the proliferation and morphology of 3D4/21 cells, we compared the cell viability after treatment with different concentrations (0, 10, 20, 30, 40, 50, and 60 μg/mL) of FB1 for different time periods (24, 48, and 72 h). The results showed that FB1 decreased the viability of 3D4/21 cells and promoted cell damage in a time-dependent manner. When treated with 50 μg/mL FB1 for 24 h, cell viability decreased to 60.1%, and reduced cell fusion, longer and shrunken cells and loss of normal cell morphology were observed (Figure 1A,B). We next examined α-Tubulin, which is an important component of the eukaryotic cytoskeletal system and known to be crucial for cell morphology, cell division and signal transduction [24]. Immunofluorescence imaging showed that FB1 exposure resulted in a significant decrease in α-Tubulin protein (Figure 1C). This condition was selected for subsequent experiments. We found that expression of cell cycle-related genes *Cyclin D1/E1* was significantly upregulated and *CDK2/4* and *PCNA* expression was significantly reduced following FB1 exposure (Figure 1D). The effect of FB1 on apoptosis was examined using the Annexin V/FITC/PI apoptosis kit. Compared with the control group, the apoptotic rate was significantly higher after FB1 treatment (Figure 1E,F). The expression levels of various apoptosis-related genes (*Caspase-3*, *Caspase-8* and *Caspase-9*) and inflammation-related genes (*IL-6*, *IL-1β*, *TNF-α* and *IFN-γ*) were significantly upregulated, and the Bcl2/Bax ratio was significantly reduced after FB1 induction (Figure 1G,H). Together, these results showed that FB1 could lead to significant cytopathic effects: the alteration of the cell cycle and exacerbation of apoptosis and inflammation.

### 3.2. FB1 Induces Oxidative Stress and Mitochondrial Damage

**Figure 2:**
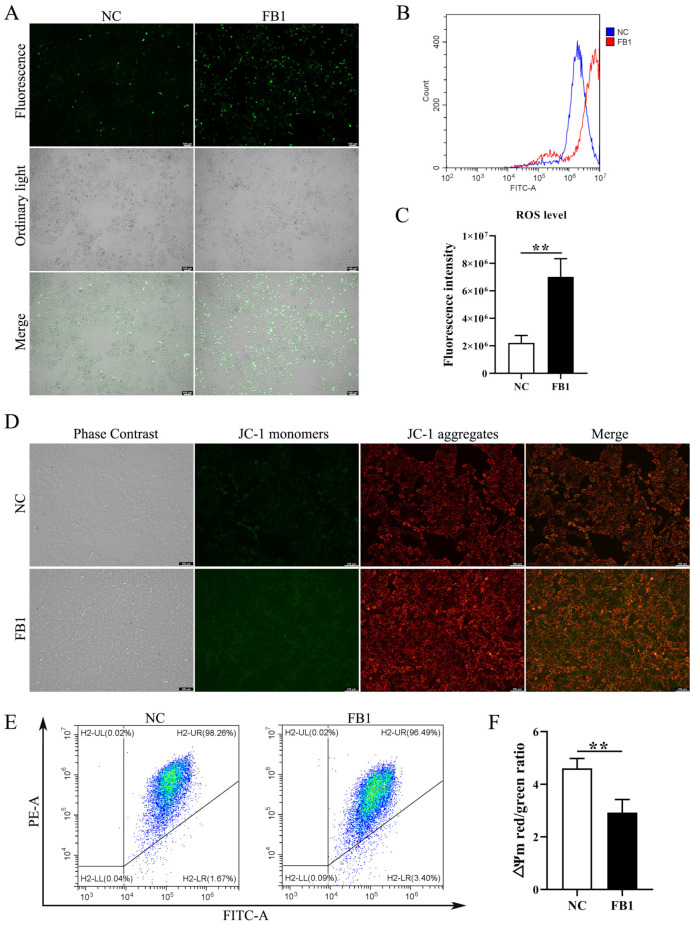
Intracellular ROS level and mitochondrial membrane potential in 3D4/21 cells induced by FB1. (**A**) Fluorescence images of intracellular ROS in 3D4/21 cells under FB1 induction. Scale bar: 100 µm. (**B**) The intracellular ROS levels analyzed by flow cytometry; (**C**) Quantification of (**B**). (**D**) Fluorescence map of mitochondrial membrane potential in 3D4/21 cells exposed to FB1. Scale bar: 100 µm. (**E**) Detection of mitochondrial membrane potential by flow cytometry. (**F**) The ratio of red fluorescence intensity to green fluorescence intensity measured by flow cytometry (ΔΨm). NC: (3D4/21 cells without FB1 exposure), FB1 (50 μg/mL FB1 for 24 h). Data are presented as the mean ± SD; ** *p* < 0.01.

The changes in intracellular basic ROS level and mitochondrial membrane potential (∆Ψm) are important indicators of the functional status of healthy mitochondria. We examined changes in ROS levels following FB1 treatment of 3D4/21 cells, and found ROS fluorescence signals were significantly enhanced in the FB1 exposed group (Figure 2A). Similarly, intracellular ROS levels measured by flow cytometry showed the same results (Figure 2B,C). We further determined the mitochondrial membrane potential (∆Ψm), following FB1 treatment in 3D4/21 cells, through the addition of FB1 enhanced green fluorescence compared with control (Figure 2D). We also quantified ∆Ψm changes by flow cytometry analysis of the ratio of red fluorescence intensity to green fluorescence intensity. After treatment with FB1, the ratio of red–green fluorescence intensity was significantly reduced compared with the control group (Figure 2E,F). These results indicate that FB1 can induce oxidative stress and mitochondrial damage in 3D4/21 cells.

### 3.3. PTE Attenuates FB1-Induced Damage in 3D4/21 Cells

**Figure 3:**
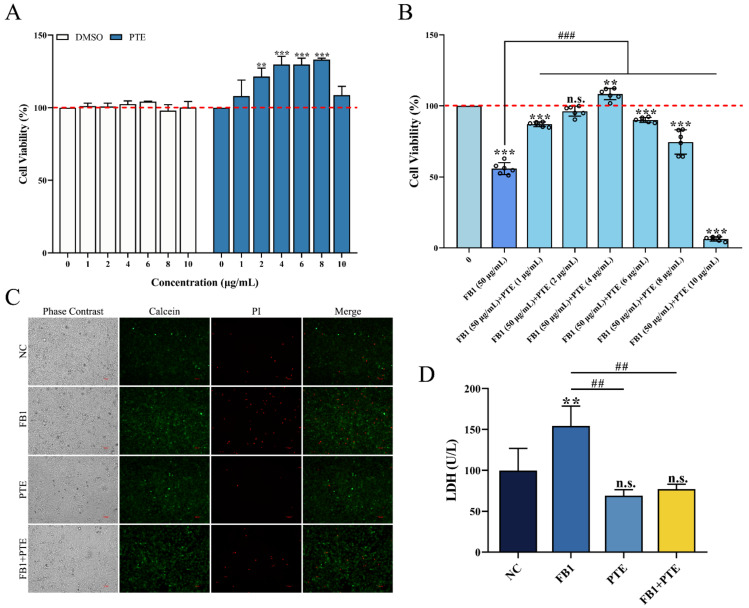
Effect of PTE on the inhibition of proliferation of 3D4/21 cells by FB1. (**A**) Effect of different gradient concentrations of PTE on the viability of 3D4/21 cells treated for 24 h. (**B**) The CCK-8 assay used to detect the effect of combined treatment with different gradient concentrations of PTE and FB1 (50 µg/mL) for 24 h on the viability of 3D4/21 cells. (**C**) Effect of PTE on the integrity of 3D4/21 cell membrane induced by FB1. Phase contrast refers to the cells observed under bright field; Calcein refers to the live cells labeled with green fluorescence; PI refers to the dead cells labeled with red fluorescence; Merge refers to the difference in fluorescence staining between the live cells and dead cells observed after superposition of Calcein and PI double staining. Scale bar: 100 µm. (**D**) Effect of PTE on lactate dehydrogenase induced by FB1 in 3D4/21 cells. NC: (3D4/21 cells without FB1 exposure), FB1 (50 μg/mL FB1 for 24 h), PTE (4 μg/mL PTE for 24 h), FB1 + PTE (50 μg/mL FB1 combined with 4 μg/mL PTE for 24 h). Data are presented as the mean ± SD. Compared with the control group, n.s., not significant; ** *p* < 0.01; *** *p* < 0.001; compared with the FB1 alone group, ## *p* < 0.01; ### *p* < 0.001.

The effect of PTE on the viability of 3D4/21 cells was evaluated by in vitro culture experiments, and the results showed that PTE did not produce cytotoxicity in 3D4/21 cells in the concentration range of 0–10 μg/mL (0, 1, 2, 4, 6, 8, and 10 μg/mL) for 24 h. Interestingly, when the concentration of PTE was in the range of 2–8 μg/mL, PTE could significantly increase the viability of 3D4/21 cells (Figure 3A). The therapeutic effect of different concentrations of PTE on FB1-induced 3D4/21 cell injury was assessed using a combination of PTE and FB1 at concentrations of 1, 2, 4, 6, 8, and 10 μg/mL; the addition of PTE significantly increased the activity of 3D4/21 cells when the PTE concentration ranged from 1 to 8 μg/mL, compared with the FB1 alone treatment group, with the highest viability of 3D4/21 cells in the PTE treatment group at a concentration of 4 μg/mL (Figure 3B). Therefore, a concentration of 4 μg/mL was selected for the PTE-treated group in subsequent experiments.

To further verify the protective effect of PTE on FB1-induced cytotoxicity in 3D4/21 cells, we found that the number of dead cells increased significantly after FB1 treatment of 3D4/21 cells by cell membrane integrity assay, while the number of cell deaths was effectively improved after treatment with a combination of PTE and FB1 (Figure 3C). The release of lactate dehydrogenase could also reflect the degree of membrane damage. Similarly, the content of LDH in 3D4/21 cells treated with the combination of PTE and FB1 was significantly lower than that of the FB1 group (Figure 3D). These results indicate that PTE is effective in improving FB1-induced cell membrane integrity and levels of lactate dehydrogenase released in 3D4/21 cells; it thereby has the potential to be applied in the reduction of FB1-induced cytotoxicity.

### 3.4. PTE Ameliorated FB1-Induced Apoptosis and Inflammation in 3D4/21 Cells

**Figure 4:**
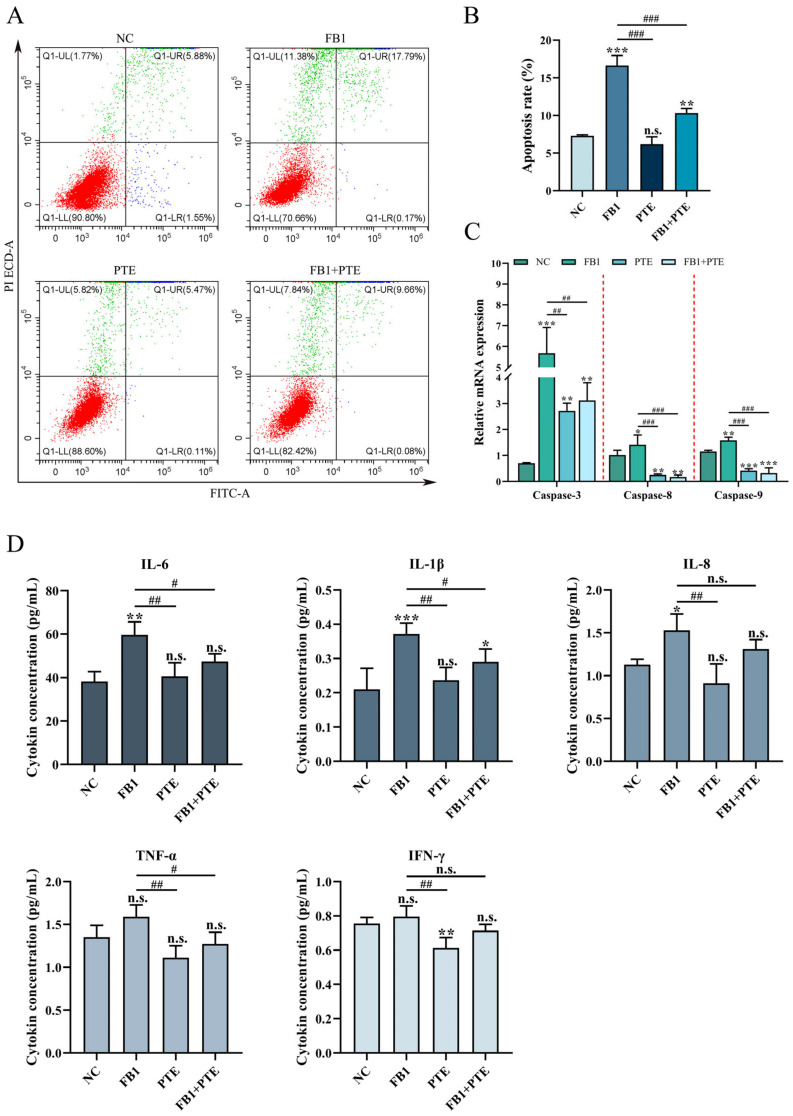
Effects of PTE on apoptosis and inflammation of 3D4/21 cells induced by FB1. (**A**) Flow cytometry analysis of Annexin V/FITC/PI stained cells. (**B**) Quantification of apoptotic rate. (**C**) qPCR analysis of expression of apoptosis-related genes. (**D**) The secretion of cytokines (IL-6, IL-1β, IL-8, TNF-α and INF-γ) in the supernatant of cell culture detected by ELISA method. NC: (3D4/21 cells without FB1 exposure), FB1 (50 μg/mL FB1 for 24 h), PTE (4 μg/mL PTE for 24 h), FB1 + PTE (50 μg/mL FB1 combined with 4 μg/mL PTE for 24 h). Data are presented as the mean ± SD. Compared with the control group, n.s., not significant; * *p* < 0.05; ** *p* < 0.01; *** *p* < 0.001; compared with the FB1 alone group, n.s., not significant; # *p* < 0.05; ## *p* < 0.01; ### *p* < 0.001.

To further determine the protective effect of PTE on FB1-induced 3D4/21 cytotoxicity, we examined the apoptosis rate of 3D4/21 cells treated with FB1, PTE and a combination of FB1 and PTE for 24 h. Combined treatment with FB1 and PTE significantly decreased the apoptosis rate compared with the FB1-treated group (Figure 4A,B), and combined treatment with FB1 and PTE significantly decreased the expression of apoptosis-related genes *Caspase-3*, *Caspase-8* and *Caspase-9* (Figure 4C). In addition, we also examined the secretion levels of cytokines associated with inflammation, and the combined treatment of FB1 and PTE significantly improved the secretion levels of IL-6 and IL-1β compared with the FB1 group (Figure 4D). Taken together, our results suggest that PTE treatment ameliorates FB1-induced apoptosis with inflammation.

### 3.5. PTE Ameliorates the FB1-Induced Oxidative Stress and Mitochondrial Damage in 3D4/21 Cells

**Figure 5:**
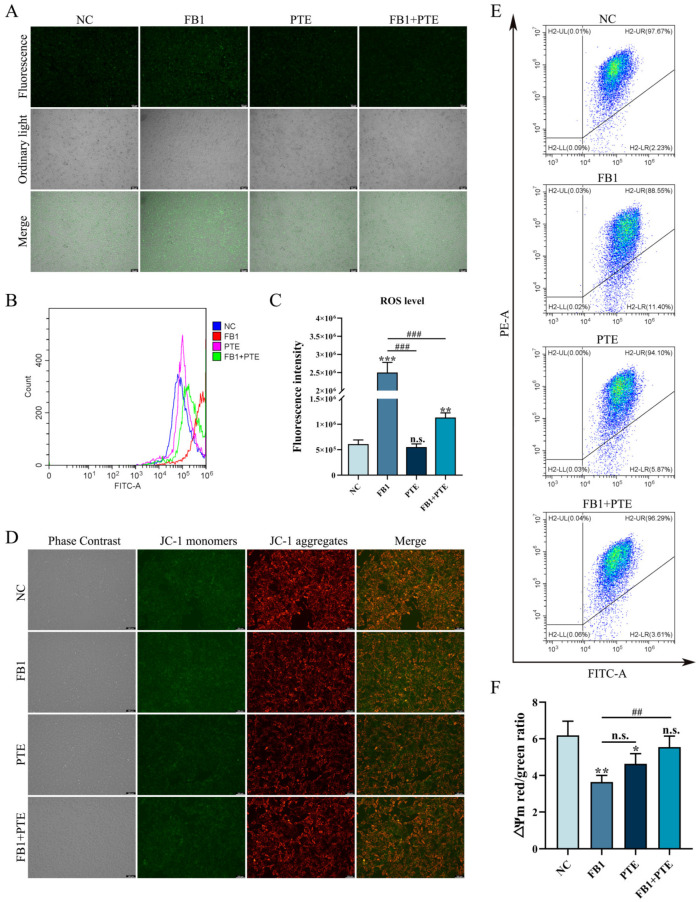
Effect of PTE on ROS levels and mitochondrial membrane potential induced by FB1 in 3D4/21 cells. (**A**) Representative fluorescence images of intracellular ROS in 3D4/21 cells. Scale bar: 100 µm. (**B**) Intracellular ROS levels analyzed by flow cytometry. (**C**) Fluorescence intensity quantification of intracellular ROS in different groups of 3D4/21 cells. (**D**) Fluorescence map of intracellular mitochondrial membrane potential in 3D4/21 cells. Scale bar: 100 µm. (**E**) Detection of mitochondrial membrane potential by flow cytometry. (**F**) The ratio of red fluorescence intensity to green fluorescence intensity measured by flow cytometry (ΔΨm). NC: (3D4/21 cells without FB1 exposure), FB1 (50 μg/mL FB1 for 24 h), PTE (4 μg/mL PTE for 24 h), FB1 + PTE (50 μg/mL FB1 combined with 4 μg/mL PTE for 24 h). Data are presented as the mean ± SD. Compared with the control group, n.s., not significant; * *p* < 0.05; ** *p* < 0.01; *** *p* < 0.001; compared with the FB1 alone group, n.s., not significant; ## *p* < 0.01; ### *p* < 0.001.

Oxidative stress is an important mechanism of FB1 toxicity, and we examined the effect of PTE on the level of ROS induced by FB1 in 3D4/21 cells. Compared with the control group, 3D4/21 cells treated with FB1 alone could significantly increase the intracellular ROS fluorescence intensity, while the addition of PTE alone had no effect on the intracellular ROS fluorescence intensity of 3D4/21 cells. However, compared with FB1 group, the combination of PTE and FB1 could weaken the intracellular ROS fluorescence intensity of 3D4/21 cells (Figure 5A). In addition, analysis of relative fluorescence intensity of ROS by flow cytometry showed that the addition of PTE significantly decreased the level of ROS production compared with the FB1 group (Figure 5B,C). To further verify the protective effect of PTE on mitochondria in 3D4/21 cells exposed to FB1, ∆Ψm values were measured in 3D4/21 mitochondria after PTE treatment. The results showed that the addition of FB1 increased the green fluorescence level compared with the control and PTE groups, while the addition of PTE decreased the green fluorescence level compared with the FB1 group (Figure 5D). In parallel, we quantified changes in ∆Ψm by the ratio of red fluorescence intensity to green fluorescence intensity (Figure 5E,F). These results indicate that PTE ameliorates FB1-induced oxidative stress and mitochondrial damage in 3D4/21 cells.

### 3.6. PTE Inhibits FB1 Activated JAK/STAT Pathway

**Figure 6:**
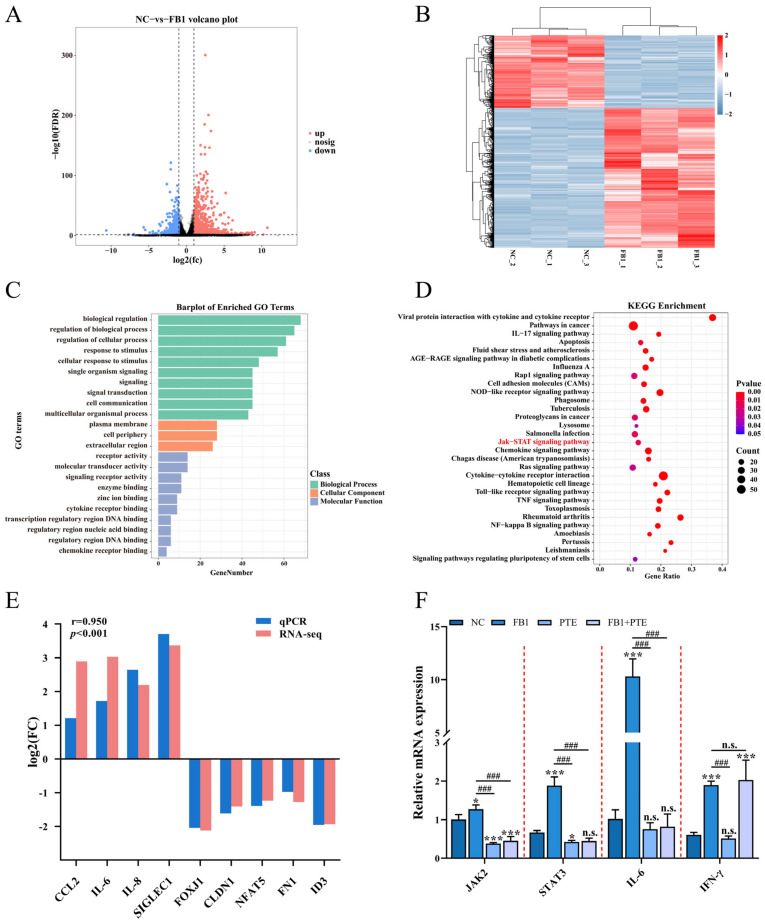
FB1 exposure leads to altered expression of hundreds of genes in 3D4/21 cells. (**A**,**B**) Volcano plot and heatmap of differentially expressed genes in 3D4/21 cells following exposure to FB1. (**C**,**D**) GO enrichment analysis and KEGG enrichment analysis of differentially expressed genes. (**E**) qPCR validation of differentially expressed genes. (**F**) PTE can downregulate FB1-induced expression of genes important for JAK/STAT signaling pathway. NC: (3D4/21 cells without FB1 exposure), FB1 (50 μg/mL FB1 for 24 h), PTE (4 μg/mL PTE for 24 h), FB1 + PTE (50 μg/mL FB1 combined with 4 μg/mL PTE for 24 h). Data are presented as the mean ± SD. Compared with the control group, n.s., not significant; * *p* < 0.05; *** *p* < 0.001; compared with the FB1 alone group, n.s., not significant; ### *p* < 0.001.

Specific molecular mechanisms need to be elucidated after demonstrating that FB1 can destroy 3D4/21 cells. We identified alterations in genome-wide gene expression following FB1 treatment of 3D4/21 cells using RNA-Seq, and identified a total of 1536 differentially expressed genes (DEGs), including 1012 upregulated and 524 downregulated (Figure 6A,B and Table S3). GO enrichment analysis showed that FB1 treatment mainly affected biological processes such as cellular response to stimulus, signal transduction and lipid transport (Figure 6C and Table S4). Meanwhile, KEGG pathway analysis revealed that these DEGs were closely involved in the JAK/STAT signaling pathway, cytokine–cytokine receptor interaction, and metabolic pathways (Figure 6D and Table S5). Ten DEGs (*CCL2*, *IL-6*, *IL-8*, *SIGLEC1*, *FOXJ1*, *CLDN1*, *NFAT5*, *FN1* and *ID3*) were randomly selected and confirmed by qPCR (Figure 6E). Notably, the JAK/STAT signaling pathway is a member of the intracellular signal transduction pathway family [25], and previous studies have shown that increases in cytokines, such as IL-6 and TNF-α, in macrophages are closely related to activation of the JAK/STAT signaling pathway [26]. To further investigate the mechanism by which PTE attenuates cytotoxicity through the JAK/STAT signaling pathway, qPCR was used to detect the expression of *JAK2*, *STAT3*, *IL-6* and *INF-γ*, important genes of the JAK/STAT signaling pathway; the results showed that the expression levels of JAK2, STAT3 and IL-6 were significantly lower in the FB1 + PTE group than in the FB1 alone group (Figure 6F).

## 4. Discussion

As an important mycotoxin commonly detected in corn and other diets, the toxicity of FB1 has received increasing attention in recent years. Because it is widely found in food and feed, farm animals, especially pigs, are easily exposed to FB1. Porcine pulmonary edema can occur in pigs ingesting FB1-contaminated feed, which has not been found in other species following exposure to FB1, this change appears to be specific to pigs [27,28]. Alveolar macrophages distributed in the alveolar space are the most abundant innate immune cells in the distal parenchyma of the lung, which orchestrate the initiation and breakdown of pulmonary immune responses and provide efficient non-specific defense for lung tissue [29]. However, the direct toxic effect of FB1 on 3D4/21 cells and its mechanism are not clear.

In this study, we examined the survival rate of 3D4/21 cells induced by different concentrations of FB1 with in vitro experiments of different duration, and determined that 3D4/21 cell activity showed a decreasing trend with increasing FB1 concentration and induction time. Apoptosis is important for maintaining the balance between various systems in organisms, and an abnormal increase in apoptosis can adversely affect organisms; furthermore, the apoptosis rate of 3D4/21 cells is significantly upregulated after FB1 stimulation. Caspases are common pathways of both extrinsic (death receptor pathway) and intrinsic (mitochondrial pathway) apoptosis signal transduction pathways [30]. Caspase-9 acts as a mammalian apoptosis promoter, and when 3D4/21 cells sense the stimulation of the harmful substance FB1, their expression increases and transmits the signal to the downstream apoptotic executioner Caspase-3, which changes 3D4/21 cell membrane permeability, leading to 3D4/21 cell apoptosis [31]. The mRNA expression and secretion of inflammatory cytokines IL-6 and IL-1β were significantly increased after the 3D4/21 cells were induced by FB1. Immunostimulation or immunosuppression induced by FB1 depends on the concentration and duration of action. When FB1 acts on 3D4/21, cells will prompt cells to produce cytokines that regulate immunity to improve immune function, to counteract the damage caused by FB1. The mRNA expression level of inflammatory cytokines in 3D4/21 cells is significantly upregulated after FB1 induction, indicating that FB1 can induce 3D4/21 cells to produce inflammation. Changes in intracellular ROS levels are important indicators of cellular functional status, as ROS belongs to metabolites produced in normal cells of the body [32]. Normally, moderate intracellular ROS is associated with biological processes such as cell proliferation and modification of post-translational proteins, while excessive ROS can trigger intracellular oxidative stress, leading to apoptosis [33,34]. We detected that FB1 induction significantly elevated ROS levels in 3D4/21 cells, indicating that 3D4/21 cells were under increased damage by oxidative stress. The function of mitochondria is related to ATP production, ROS production, cytoplasmic redox regulation and apoptosis [35,36]. However, excessive ROS disrupts the mitochondrial membrane, allowing ions inside and outside the membrane to reduce the concentration difference by free diffusion, which leads to a decrease in mitochondrial membrane potential, triggering oxidative stress and impairing mitochondrial function [37]. FB1 disrupts the balance of intracellular ROS and antioxidant capacity, leading to mitochondrial dysfunction.

Polyphenols are a class of natural compounds present in plants, and have potential health-promoting effects [38]. Polyphenols in plants possess various protective properties such as antimicrobial, anti-inflammatory, antioxidant and anticancer properties [39]. Natural plant extract resveratrol, as a polyphenol, has been demonstrated to be effective in improving aflatoxin B1 damage to bovine mammary epithelial cells [40]. As a natural analogue of resveratrol, PTE has anti-inflammatory, antioxidant and neuroprotective effects similar to resveratrol [18,41], while PTE has higher bioavailability and chemical stability compared with resveratrol [42]. In a co-culture model of 3T3-L1 adipocytes and RAW 264.7 macrophages, PTE significantly decreased the secretion of cytokines IL-6 and TNF-α, while significantly downregulating the mRNA expression levels of pro-inflammatory genes *COX-2*, *IL-6* and *TNF-α*, and also reducing macrophage migration to adipocytes [43]. Cell necrosis is the form of cell death observed in disease pathology which leads to the release of plasma membrane ruptured cellular contents into the extracellular environment; necrosis can also cause an inflammatory response [44]. Oxidative stress and inflammatory response are important factors leading to cell damage and apoptosis [45]. The increase in ROS and imbalance of the antioxidant defense system can lead to mitochondrial damage in cells, while PTE can significantly reduce ROS production after acting on FB1-induced 3D4/21 cells, and this protective effect may be derived from the ability of PTE to effectively regulate antioxidant enzyme levels, to inhibit the production of oxidative free radicals and to reduce the loss of mitochondrial membrane potential, which in turn helps to reduce FB1-induced cell damage [15]. The Caspase family is an important regulator of apoptosis [46]. Caspase-3 catalyzes the cleavage of many key cellular proteins, and is essential for chromatin condensation and DNA degradation of all apoptosis [47]. The results of this study showed that the expression levels of *Caspase-3* and *Caspase-9* genes were significantly higher after FB1 treatment in 3D4/21 cells, while PTE co-treatment with FB1 effectively inhibited the expression of Caspase-3 and Caspase-9. These results suggest that PTE may attenuate FB1-induced apoptosis by inhibiting the expression of certain apoptosis-related genes.

To further validate the mechanism of the cytotoxic mitigation effect of PTE on FB1 production, we used RNA-seq to select the possible pathway of FB1-induced 3D4/21 cytotoxicity, the JAK/STAT signaling pathway. As an important intracellular signal transduction pathway, the JAK/STAT signaling pathway is associated with apoptosis, induction of inflammation, control of immune response and oxidative stress [48,49]. Increases in cytokines are closely associated with activation of the JAK/STAT signaling pathway in macrophages [50]. Aflatoxin B1 can induce immunotoxicity in 3D4/21 cells via the DNA methyltransferase-mediated JAK2/STAT3 pathway [51]. In this study, we verified that PTE had a significant effect on FB1-induced expression of *JAK2*, *STAT3*, *IL-6* and *INF-γ*important genes of the JAK/STAT signaling pathway according to which we concluded that the JAK/STAT signaling pathway may serve as a key pathway for PTE to alleviate FB1 cytotoxicity. However, transcriptome data show that there are dozens of key pathways that may be regulated by FB1, and we did not analyze the function of other key pathways (such as the NF-kappa B signaling pathway) which may also be worth studying in the future.

## 5. Conclusions

**Figure 7:**
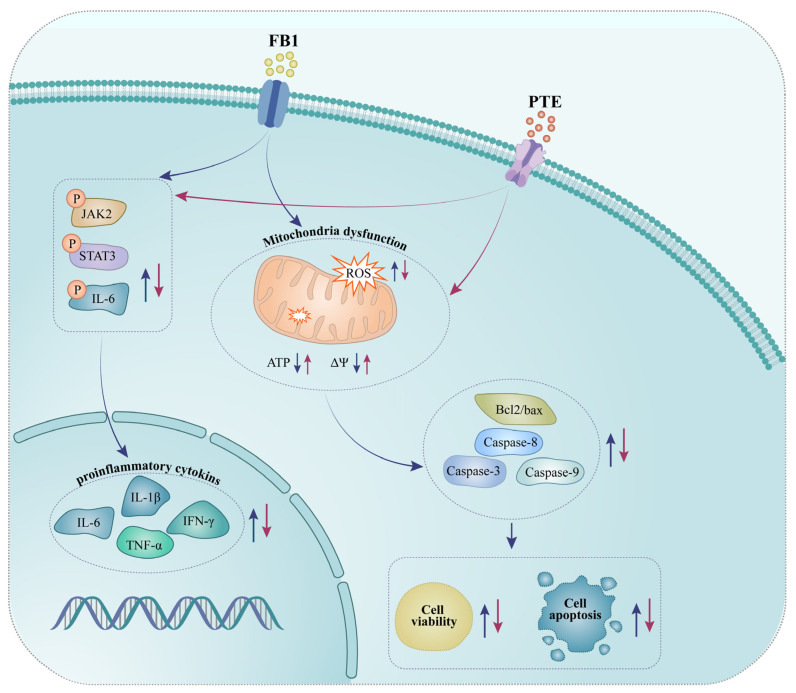
Schematic representation of exposure of porcine alveolar macrophages to FB1 and pterostilbene addition.

Taken together, these results indicate that FB1 exposure induced oxidative stress and inflammation-mediated apoptosis, and altered mRNA expression of inflammatory genes in 3D4/21 cells. PTE significantly protected 3D4/21 from FB1 toxicity through its antioxidant and anti-inflammatory effects. Mechanistically, PTE may mitigate the toxic effects of FB1 on 3D4/21 by participating in the JAK/STAT signaling pathway (Figure 7). Our study found PTE to be a potential drug to alleviate diseases caused by FB1 contamination.

## Data Availability

The RNA-seq data generated in this study have been deposited in the NCBI Gene Expression Ominibus (GEO) database under the accession number GSE190291. Gene Expression Omnibus. https://www.ncbi.nlm.nih.gov/geo/query/acc.cgi?acc=GSE190291 (accessed on 23 October 2022). (The following secure token has been created to allow review of record GSE190291 while it remains in private status: slurgooulfwnbih).

## Supplementary Materials

The following supporting information can be downloaded at: https://www.mdpi.com/article/10.3390/antiox11122360/s1, Table S1: Primer sequences used in qPCR assays; Table S2: Primer sequences used in qPCR assays for differentially expressed genes; Table S3: Differentially expressed genes between control groups and FB1 treatment group; Table S4: Functional enrichment analysis for differentially expressed genes; Table S5: Pathway analysis of differential metabolites between control groups and FB1 treatment group (XLSX).
